# The importance of blood parameters in the detection of intestinal metaplasia and early diagnosis of gastric cancer

**DOI:** 10.4314/ahs.v24i3.25

**Published:** 2024-09

**Authors:** Ayşe Kefeli, Feyyaz Onayrılı, Fahri Gokcal

**Affiliations:** 1 Tokat Gaziosmanpasa University, Gastroenterology Department, Gaziosmanpasa, Tokat, Turkiye; 2 Tokat Gaziosmanpasa University, Internal Medicine, Gaziosmanpasa, Tokat, Turkiye; 3 Good Samaritan Medical Center, Tufts University School of Medicine, Surgery department, Boston, MA, USA

**Keywords:** Gastric cancer, intestinal Metaplasia, Monocyte to lymphocyte ratio, red cell distribution, hemoglobin, platelet, neutrophil-lymphocyte ratio, platelet-lymphocyte ratio, inflammation

## Abstract

**Background:**

We aimed to determine whether complete blood count(CBC) parameters, such as the white blood count(WBC), hemoglobin(Hb), platelet(PLT), red cell distribution width(RDW), mean platelet volume(MPV), platelet distribution width(PDW), neutrophil-to-lymphocyte ratio(NLR), platelet-to-lymphocyte ratio(PLR), and monocyte-to-lymphocyte ratio(MLR), have a predictive value in the detection of gastric cancer(GC) and intestinal metaplasia(IM).

**Methods:**

While proven GC, IM, and healthy control(HC) patients were included, patients with the comorbid disease were excluded, and univariate analyses compared three groups. The receiver operating characteristic(ROC) curve analysis was run for CBC parameters. The area under the curve(AUC) was evaluated, and a cut-off value was determined. The sensitivity and specificity of each parameter were considered.

**Results:**

The GC, IM, and HC groups consisted of 72(33%), 73(34%), and 72(33%) patients, respectively. RDW, PLT, NLR, PLR, and MLR were significant between GC and IM. The highest AUC (0.727) was obtained for the PLT yielding a 56.9% sensitivity and 79.4% specificity at a cut-off value of 151.8. The AUC of RDW was found as 0.691 and 0.626 for pairwise comparisons of GC-HC and IM-HC, respectively. At a cut-off value of 13.4, PLR yielded 70.8% sensitivity and 61.1% specificity in the detection of GC, while 64.4% sensitivity and 51.1% specificity for IM.

**Conclusion:**

CBC parameters, such as RDW, PLT, NLR, PLR, and MLR, have value in detecting GC. RDW also has diagnostic value in helping to detect IM. PLR can help to distinguish patients with GC from those with IM. These inexpensive, easily accessible parameters may help in the timely diagnosis of GC and IM.

## Introduction

Gastric cancer (GC) is an insidious disease with late manifestations. GC is the 4th most common cancer worldwide (7.8% of cancers) and the third leading cause of cancer related death (9.7%) 1. Since the prognosis of gastric carcinoma depends on the stage of diagnosis, late diagnosis may lead to an increase in mortality and morbidity. Regarding symptomatology, the most common initial symptom is a vague feeling of discomfort and fullness in the epigastrium. Consequently, the disease is often at an advanced stage when diagnosed.

Clinical and epidemiological studies showed that inflammation is crucial in the pathogenesis of GC^2^. Intestinal metaplasia (IM) is considered a precancerous condition and one of the histopathological changes in the inflammatory cascade, known as Correa^3^. IM independently confers risk for the development of GC^4^. Therefore, diverse efforts have been made to detect premalignant or malign gastric lesions as early as possible.

The current guideline recommends screening the people at risk may decrease GC mortality by allowing early detection and treatment, often by endoscopy instead of more invasive surgery. However, routine endoscopic screening of patients with non-specific symptoms in intermediate or low-incidence countries is not recommended due to its cost-effectiveness and invasiveness^4^. In order to detect GC or premalignant lesions with endoscopy, which patient requires screening should be determined with easily accessible and applicable tests. Screening for the disease with low-cost and simple blood tests may be a reasonable option^5^. For this purpose, several biomarkers have been studied to determine who should be screened first, with or without alarm symptoms. Also, various parameters obtained from the complete blood count (CBC) test, which is easily accessible, inexpensive, and practical, have been studied. Some parameters, such as red cell distribution width (RDW), mean platelet volume (MPV), neutrophil/lymphocyte ratio (NLR), and platelet/lymphocyte ratio (PLR), have been found as associated with tumor development, progression, and survival^2^, ^3^ ^6^. MPV of these, has been suggested as an inflammatory marker in various inflammatory diseases, malignity particularly in gastric cancer^2^ and also including nasal polyposis^7^, rheumatoid arthritis^8^ obesity^9^ infections^10^, and type 2 diabetes mellitus^11^. In addition, RDW is linked with inflammation in thyroiditis^12^, autoimmune hepatitis^13^, and chronic kidney disease^14^. Elevated RDW levels also have been associated with gastric cancer^15^,^16^. Looking at the literature, several studies has revealed a significant association between NLR and inflammatory diseases such as Inflammatory Bowel Disease(IBD)^17^ Hashimoto's disease^18^, NAFLD^19^, thyroid conditions^20^, COVID-19 21, and survival of the patients with gastric cancer^22^. Similarly, PLR was suggested as a marker of inflammation in pancreatitis^23^, IBD^24^, type 2 diabetes mellitus^25^, cancer^26^ and gastric cancer^27^. Lastly, MLR, is another hemogram-derived marker, considered an inflammatory predictor in diabetic nephropathy^28^, IBD^29^, malignancy^30^, and gastric cancer^31^. However, the literature on using of these parameters in the early detection of GC and IM is still scarce^32^.

Therefore, in this study, we aimed to determine whether CBC parameters, such as the white blood count (WBC), hemoglobin (Hb), platelet (PLT), RDW, MPV, Platelet Distribution Width (PDW), NLR, PLR, Monocyte Lymphocyte Ratio (MLR), have a predictive value in the detection of GC and IM.

## Materials and Method

We retrospectively reviewed the patients who underwent an upper gastrointestinal system endoscopic biopsy at a tertiary health center between 1 June 2014 and 15 December 2021. Patients who had a reported histopathological result of GC or IM and those who did not have GC or IM were selected for this study.

Patients with the following conditions were excluded from the study; hypertension, diabetes mellitus, antiplatelet or anti-inflammatory treatment, hyperlipidemia, thyroid diseases, autoimmune diseases, rheumatological diseases, heart disease, chronic liver disease, chronic kidney disease, and those with a previous history of malignancy. Statistical analyses were run on the GC, IM, and HC patient groups.

Patient demographics (age, gender) and the following CBC parameters were recorded; the WBC, Hb, neutrophil, lymphocytes, monocyte, PLT, RDW, MPV, and PDW. An NLR, PLR, and MLR values were calculated separately as the absolute counts of neutrophils, platelets, and monocytes divided by the absolute lymphocytes count.

The CBC results were obtained before the endoscopic biopsy was used for this study. Venous blood samples were drawn into ethylenediamine tetraacetic acid (EDTA) tubes (5 mL). All samples were processed within 2 hours after vein puncture using an automated hematology analyzer in our hospital. Normal values for studied blood parameters ranged as follows: (WBC): 4.6–10.2 × 103/µl, neutrophil: 2.0–6.9 × 103/µl lymphocyte: 0.6–3.4 × 103/µl monocyte: < 0.9 × 103/µl, hb:12–15.4 g/dl, platelet: 142–424 × 103/µl MPV: 7.8–11 fl, RDW: 11.6–17.2%, PDW: 8.3–25 fl.

### Statistical analysis

Statistical assessments were performed using SPSS (Statistical Package for Social Sciences for Windows, Version 25.0, Armonk, NY, USA: IBM Corp.). Qualitative data were expressed as frequency and percentage [n (%)], and quantitative data were expressed as mean and standard deviation (SD), based on their distribution that assessed by running Kolmogorov-Smirnov and Shapiro-Wilk tests. The Chi-square test was used for qualitative variables, One Way ANOVA (F test) was used for quantitative variables, and The post hoc Tukey HSD test was used for pairwise comparisons. The receiver operating characteristic (ROC) curves were performed; the area under the curve (AUC) and 95% confidence interval were calculated for the studied CBC parameters. The sensitivity and specificity values were calculated according to cut-off points identified as the value closest to 1 in the ROC curves. A p-value <0.05 was considered significant.

The data were entered into the G-Power package program to calculate the study's sample size. The total sample size was calculated as at least 189 individuals, with a power of 95% and a Type I error (alpha) of 0.05.

## Results

After inclusion and exclusion criteria were applied to 3000 patients who underwent upper gastrointestinal system endoscopic biopsy, two hundred seventeen patients were included in this study; the GC, IM, and HC groups consisted of 72(33%), 73(34%), and 72(33%) patients, respectively. The comparison of patient demographics and CBC parameters is given in Table 1. There was no difference between the groups in terms of gender (p= 0.843). The average age of the GC group was higher than those in the HC group in the pairwise comparison (p= 0.015).

**Table 1:** Demographic characteristics and laboratory parameters across the study groups

	Gastric Cancer (n=72)	Intestinal Metaplasia (n=73)	Healthy Control (n=72)	f	p	Pairwise Comparison
Age, year, mean ± SD	60.47 ± 10.5	57.5 ± 10.3	55.1 ± 12.2	4.292	**0.015**	GC-HC
Sex, male, n (%)	38 (52.8)	35 (47.9)	36 (50)	0.341	0.843	-
WBC, ×10^3^/µl, mean ± SD	7.9 ± 3.3	7.1 ± 1.8	7.3 ± 2	2.409	0.092	-
Hb, g/dl, mean ± SD	12.2 ± 2.4 5	13.4 ± 2	13.7 ± 1.9	10.108	**<0.001**	GC-IM, GC-HC
PLT, ×10^3^/µl, mean ± SD	314.7 ± 103.6	260.9 ± 63	267.3 ± 58.2	10.184	**<0.001**	GC-IM, GC-HC
RDW, %, mean ± SD	15.4 ± 3.3 3	14.5 ± 2.3	13.7 ± 2.1	6.939	**0.001**	GC-HC, IM-HC
MPV, fl, mean ± SD	8.9 ± 1.4	9.3 2 ± 1.1 2	9.2 ± 1.3	1.307	0.273	-
PDW, %, mean ± SD	14.4 ± 3.4 3	14.7 ± 3.4	14.5 ± 3.4	0.051	0.951	-
NLR, mean ± SD	2.9 ± 2	2.1 ± 1.3	2.2 ± 1.24	5.808	**0.003**	GC-IM, GC-HC
PLR, mean ± SD	177.1 ± 76.3	123.8 ± 57.7	132.7 ± 55.5	14.409	**<0.001**	GC-IM, GC-HC
MLR, mean ± SD	0.34 ± 0.19	0.27 ± 0.12	0.289 ± 0.16	3.548	**0.030**	GC-IM, GC-HC

*WBC* White Blood Cell, *Hb* Hemoglobin, *PLT* Platelet, *RDW* Erythrocyte Distribution Width, *MPV* Mean Platelet Volume, *PDW* Platelet Distribution Width, *NLR* Neutrophil Lymphocyte Ratio, *MLR* Monocyte Lymphocyte Ratio, *PLR* Platelet Lymphocyte Ratio, *f* One-Way Analysis of Variance (ANOVA) The post hoc Tukey HSD test was used for pairwise comparisons.

When gastric cancer, IM, and HC groups were compared with the Post Hoc Tukey HSD test, there was no difference between paired comparison groups regarding WBC, MPV, and PDW. There was a difference in RDW between GC and HC groups, higher in GC and between IM and HC groups in IM. For Hb, PLT, NLR, PLR, and MLR, there was a difference between GC and IM and GC and HC groups, with a higher difference in GC.

**Figure 1:**
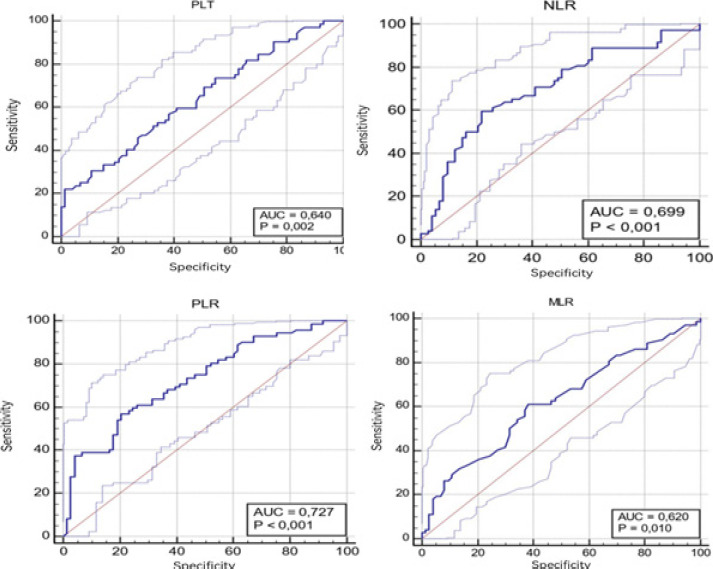
ROC analysis curves of complete blood count parameters between Gastric Cancer-Intestinal Metaplasia Groups (*AUC* Area under the curve, *PLT* Platelet, *NLR* Neutrophil Lymphocyte Ratio, *MLR* Monocyte Lymphocyte Ratio, *PLR* Platelet Lymphocyte Ratio)

The ROC curve analysis results for the CBC parameters of GC and IM groups are presented in Table 2 and Figure 1. When we used the cut-off value of 154 for the PLT, the sensitivity was calculated as 22.2%, and the specificity was 98.6% (AUC: 0.64). When the cut-off value for NLR was taken as 2.28, the sensitivity was calculated as 59.7%, and the specificity was 78.1% (AUC: 0.69). When the cut-off value for PLR was taken as 151.8, the sensitivity was 56.9%, and the specificity was 79.5% (AUC: 0.72). When the cut-off value for MLR was taken as 0.027, the sensitivity was calculated as 61.1%, and the specificity was 61.6% (AUC: 0.62).

**Table 2:** The receiver operating characteristic (ROC) curve results for the complete blood count parameters between gastric Cancer-Intestinal Metaplasia groups

Parameters	Cut-Off	AUC	SE	95% CI	Sensitivity (%)	Specificity (%)	p
RDW,%	14.6	0.581	0.0479	0.496-0.662	51.39	71.23	0.091
PLT,x103/µl	154	0.640	0.0457	0.556-0.718	22.22	98.63	**0.002**
NLR	2.28	0.699	0.0442	0.617-0.772	59.72	78.08	**<0.001**
PLR	151.8	0.727	0.0417	0.647-0.798	56.94	79.45	**<0.001**
MLR, %,	0.027	0.620	0.0467	0.535-0.669	61.11	61.64	**0.010**

*AUC* Area under the curve, *SE* Standard error, *CI* Confidence Interval, *PLT* Platelet, *NLR* Neutrophil Lymphocyte Ratio, *PLR* Platelet Lymphocyte Ratio, *MLR* Monocyte Lymphocyte Ratio

**Figure 2:**
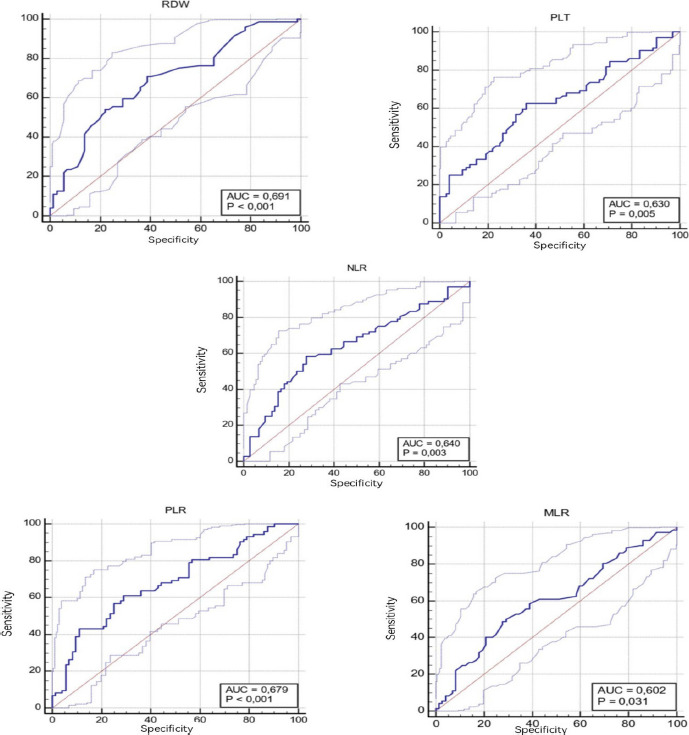
ROC analysis curves of complete blood count parameters between gastric cancer and healthy control Groups (*AUC* Area under the curve, *RDW* Erythrocyte Distribution Width, *PLT* Platelet, *NLR* Neutrophil Lymphocyte Ratio, *PLR* Platelet Lymphocyte Ratio, *MLR* Monocyte Lymphocyte Ratio)

The ROC curve analysis results for the CBC parameters of GC and HC groups are given in Table 3A and Figure 2. When the cut-off value for RDW was taken as 13.4, the sensitivity was calculated as 70.8% and the specificity as 61.1% (AUC: 0.691). When the cut-off value for PLT was taken as 275.7, the sensitivity was calculated as 62.5% and the specificity as 63.8% (AUC: 0.63). When the cut-off value for NLR was taken as 0.305, the sensitivity was calculated as 58.33% and the specificity as 72.2% (AUC: 0.64). When the cut-off value for PLR was taken as 143.6, the sensitivity was calculated as 61.1% and the specificity as 70.8% (AUC: 0.67). When the cut-off value for MLR was taken as 0.3, the sensitivity was calculated as 50% and the specificity as 70.8% (AUC: 0.602).

**Table 3. A-):** Cut-off values of complete blood count parameters between gastric cancer and healthy control groups

Parameters	Cut-Off	AUC	SE	95% CI	Sensitivity (%)	Specificity (%)	p
RDW,%	13.4	0.691	0.0440	0.608 - 0.765	70.83	61.11	**<0.001**
PLT, x103/µl	275.7	0.630	0.0466	0.546 - 0.709	62.50	63.89	**0.005**
NLR	0.305	0.640	0.0466	0.556 - 0.719	58.33	72.22	**0.003**
PLR	143.6	0.679	0.0447	0.596 - 0.754	61.11	70.83	**<0.001**
MLR,%	0.3	0.602	0.0474	0.518 - 0.683	50	70.83	**0.030**

**Figure 3:**
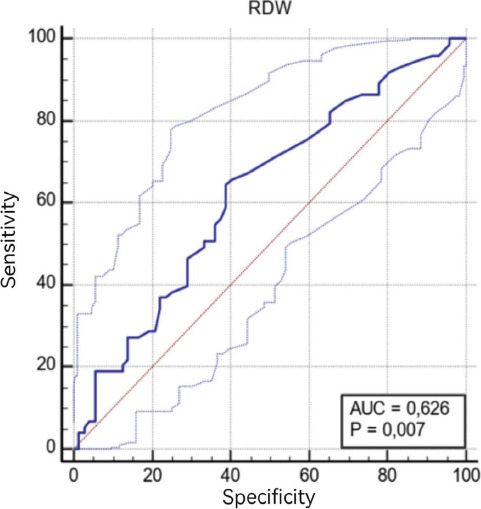
ROC analysis curve of erythrocyte distribution width between intestinal metaplasia and healthy control groups (*AUC* Area under the curve, *RDW* Erythrocyte Distribution Width)

The ROC curve analysis results for the CBC parameters of IM and HC groups are given in Table 3B and Figure 3. When we used 13.4 as a cut-off value of erythrocyte distribution width, the sensitivity was found as 64.3% and the specificity as 51.1% (AUC: 0.62).

**B-):** The cut-off value of erythrocyte distribution width between Intestinal Metaplasia-healthy control groups

Parameters	Cut-off	AUC	SE	95% CI	Sensitivity	Specificity	p
DW	13.4	0.626	0.0465	0.542-0.705	64.38	51.11	0.007*

*AUC* Area under the curve, *SE* Standard error, *CI* Confidence Interval, *PLT* Platelet, *NLR* Neutrophil Lymphocyte Ratio, *PLR* Platelet Lymphocyte Ratio, *MLR* Monocyte Lymphocyte Ratio

## Discussion

Recently, the most researched subject has been the relationship between malignancy and inflammation, based on the fact that cancer may be the result or cause of inflammation^3^,^33^. The lack of reliable specific tumor markers for diagnosing GC remains a challenge in providing a better prognosis for effective GC treatment based mainly on early diagnosis. This study aimed to determine the value of various hemogram parameters in the early diagnosis of GC and IM.

Cytokines and significantly IL-6 induce thrombopoietin production in hepatocytes^34^. It is thought that IL-6 is secreted locally from tumor cells and mainly by the immune system. Circulating tumor cells use PLTs as shields to escape from T and natural killer cells in the immune system, and PLTs are used to attach to the endothelium in metastatic carcinomas^35^. Thrombocytosis has been associated with poor prognosis and short survival in many malignancies such as gastrointestinal, breast, lung, ovary, and renal cell carcinoma^36^. For example, in an extensive study consists 1953 GC patients, thrombocytosis (based on over 400 thousand in this study) was seen at a rate of 6.4% and was associated with a higher T stage, lymph node positivity, and worse survival^37^. In another study conducted with 63 GC patients, thrombocytosis was found to be 11.4%, and it was associated with worse 1-year and 3-year survival. In addition, in this study, thrombocytosis was positively associated with lymph node involvement and depth of invasion^38^. In our study, similar to previous studies, the number of PLTs was higher in the GC group than in the IM and HC groups, and no statistically significant difference was found between IM and HC. In our study, which supports previous studies, a significant difference was found between GC and HC and between GC and IM in terms of thrombocytosis, but the PLT value was found to be insignificant in predicting IM.

It is well known that IL-6 can cause carcinogenesis through several signal pathways involved in carcinogenesis and metastasis. IL-6 induces the proliferation and differentiation of early progenitor cells, firstly megakaryocyte progenitors. This process causes platelet activation and aggregation^39^. Mean platelet volume and PDW are early indicators of PLT activation. Since PDW is less affected by the increase in the size of a small number of PLTs, it is more specific as an indicator of PLT activation^39^. Not only gastric cancer has been assosiatedelevated MPV, but also several neoplastic disorders have been associated with MPV^3^,^40^.

In a study including 194 patients with GC, 191 patients with gastric ulcers, and 185 control groups, it was found that MPV levels increased and PDW levels decreased in patients with gastric ulcers and in the control group compared to GC^41^. Increased MPV in gastric ulcers can be attributed to chronic inflammation, and decreased MPV in GC can be attributed to increased consumption of large PLTs^42^. However, in some studies, it has been reported that MPV is positively related to thrombopoietin and IL-6 levels, which are cytokines that regulate megakaryocyte ploidy and increase in patients with GC^2^,^43^. In our study, unlike other studies, MPV did not reveal a difference between all three groups. Differences in results can be attributed to measurement methods and sample sizes. Platelets distribution width is PLT heterogeneity and originates from megakaryocyte heterogeneity. In the aforementioned study, which included 194 patients with GC, 191 patients with gastric ulcers, and 185 control groups, PDW was found to be increased in the GC group, but its mechanism was not fully elucidated. Contrary to this study, our study did not reveal a difference between the three groups in the level of PDW, as in MPV.

Red cell distribution width is a simple hemogram parameter, an indicator of anisocytosis that measures the size heterogeneity of erythrocytes^44^. Red cell distribution width is mainly used in the differential diagnosis of iron deficiency anemia and thalassemia and to exclude iron deficiency anemia^45^. On the other hand, with more recent studies, it has been suggested that it can be used as a diagnosis or prognostic marker of various malignancies. It has been clearly shown that RDW is a reliable biomarker of cardiovascular morbidity and mortality and an indicator of oxidative stress, infections, and active, acute, and chronic inflammatory states^46^,^47^. However, there is minimal data regarding the evaluation of RDW as a biomarker of malignancy^48^. To the best of our knowledge, two studies evaluated the benefit of RDW as an additional factor in increasing the diagnostic accuracy of anemia as a screening method in colorectal cancer, and their results are inconsistent^49^,^50^. In addition, studies are showing that RDW is also valuable in determining the prognosis of several malignancies^51^,^52^. In our study, supporting the literature, RDW was higher in the GC and IM groups compared to the HC group, but there was no difference between the GC and IM groups. The reason for the enlargement of the erythrocyte distribution in malignancy is thought to be the increase in inflammation accompanied by carcinogenesis, decreased erythropoietin, decreased iron release from reticuloendothelial macrophages, and shortened erythrocyte lifespan due to related inflammatory markers. Again, these results suggest that RDW can be used to detect patients with GC and IM.

How the immune system affects the development and spread of cancer has always been one of the complex subjects to explain in immunology. Currently, the immune system plays a dual role in malignancy: it not only suppresses tumor growth by destroying or inhibiting malignant cells but also promotes tumor progression by selecting tumor cells better suited to survive in an immune-competent host. It is thought to promote tumor spread by creating conditions within the tumor microenvironment that facilitate tumor growth^53^. Although the exact mechanism is unknown, neutrophil and PLT-related inflammation and decreased lymphocyte-dependent antitumoral cellular immune response are significant in carcinogenesis. Therefore, it is known that lymphocytes decrease while neutrophils and PLT increase in patients with MC. In this context, high inflammatory markers NLR and PLR have been associated with poor prognosis in the colon, ovary, and GC. In studies on this subject, these parameters have diagnostic power and distinguish patients with GC from those without cancer^6^. Similar to these findings, in our study, there was a difference between GC and IM and GC and HC groups for NLR and PLR., but no difference between IM and HC. These results suggest that inflammatory markers such as NLR and PLR may help to detect GC but not in detecting IM.

Several studies have shown that macrophages contribute to the growth and metastasis of solid tumors 54. The macrophages adopt a trophic role, facilitating angiogenesis, matrix degradation, and tumor cell motility, which are part of the metastatic process. The macrophages also produce many compounds that may contribute to malignancy, such as mutagenic oxygen, nitrogen radicals, and some angiogenic factors^55^. Although the potential mechanisms are not fully known, monocytes inhibit the immune system by improving the tumor microenvironment through cytokines and positively affect tumor proliferation, angiogenesis, and progression. On the other hand, lymphocytes mediate cell-mediated immunity against tumors. Several studies have associated increased lymphocyte levels with a better prognosis in some solid tumors^56^,^57^. In a study of 91 patients with advanced GC, low MLR-monocytes and high lymphocyte levels were associated with easier R0 resection after neoadjuvant therapy, better prognosis, disease-free, and overall survival^58^. Increased monocyte and decreased lymphocyte counts in cancer patients may indicate decreased ability to stop tumor progression, indicating that LMR or MLR may be a good reflection of cancer progression^59^. However, there was no difference between IM and HC in our study, but it was between GC and IM and GC and HC. Therefore, it is thought that MLR may help to detect GC, but it will not help detect IM in the precursor lesion.

In the current literature, there are few studies on the detection of IM, a precursor lesion of gastric cancer, in which all three groups of GC, IM, and HC are included, so the data are limited. Kahramanoglu et al., in a study with all three groups, found that Hb levels were statistically different between all three groups. Mean WBC, NLR, PLR, and MLR levels were different between GC and IM groups, and RDW, WBC, MPV, PDW, NLR, PLR, and MLR levels were different between GC and HC groups. Erythrocyte distribution width, MPV, and PDW levels differed between IM and HC groups. Thus, it has been argued that these values may help to detect IM32. In our study, similar to the above, there was a difference between the NLR, PLR, MLR, GC, and IM groups. Unlike the above study, between gastric cancer and IM, Hb was different while PLT was no while WBC was. While hemoglobin, PLT, and RDW were no differences between the GC and HC groups in the other study, they were higher in favor of GC in our study. Like the above study, RDW was found to be different between the IM and HC groups, being higher in IM. The differences in the results show a need for more extensive studies on this subject.

In this study which was performed to determine the value of hemogram parameters in defining the risk groups in order to detect gastric cancer at an early stage, sensitivity and specificity were found to be 70.8%, 61.1% for RDW, 62.5%, 63.8% for PLT, %58.3, 72.2 for NLR, 61.1%, 70.8% for PLR, 50%, 70.8% for MLR respectively, compared to the HC group in predicting GC when ROC curve analysis was performed. Significant p values showed that these parameters could be valuable in defining the risk groups. Using these parameters may improve the cost-effectiveness by preventing evaluation unnecessary expensive techniques (gastroscopy and/or biopsy).

Our study has some limitations. First, the retrospective design made our results difficult to interpret. Second, it was single center study. Finally, last limitation was the relatively small study population. However, we think that valuable findings of present study are important to be enlisted in medical literatur.

## Conclusion

Our study showed that Hb, RDW, PLT, NLR, PLR, and MLR have diagnostic values, suggesting that these parameters can distinguish patients with GC from HCs. The increase in RDW is valuable in the prediction of GC as well as in the detection of IM. Thus, high-risk groups can be determined using simple, inexpensive, easily accessible hemogram parameters, and upper GI endoscopy can be performed on suitable patients. Herewith, the chance of curative surgery can be obtained, and patient health and health expenditures can be improved with early diagnosis.
